# Sustained abnormal bilirubin and leukocyte levels after ERCP with acute suppurative obstructive cholangitis: a case report with 2 months follow-up observation

**DOI:** 10.3389/fimmu.2026.1726277

**Published:** 2026-03-16

**Authors:** Jie Zheng, Jiahao Mo, Jintang Xiong, Jiaming He, Lu Wang, Yan Chen

**Affiliations:** 1 Department of Gastroenterology, The Second Affiliated Hospital of Guangzhou University of Chinese Medicine, Guangzhou, China; 2 The Second Clinical College of Guangzhou University of Chinese Medicine, Guangzhou, China; 3 Department of Radiology, The Second Affiliated Hospital of Guangzhou University of Chinese Medicine, Guangzhou, China

**Keywords:** acute suppurative obstructive cholangitis, case report, eosinophilia, liver fluke infection, refractory post-ERCP inflammation

## Abstract

Acute Suppurative Obstructive Cholangitis (AOSC) typically resolves post-ERCP. However, a 53-year-old male had persistent hyperbilirubinemia and leukocytosis (WBC 28.12×10^9^/L) along with marked eosinophilia (20.26×10^9^/L) despite standard antibiotic therapy. Systematic diagnosis ruled out residual calculi, Endoscopic Nasobiliary Drainage (ENBD) dysfunction, biliary neoplasms, and sclerosing cholangitis. A history of undercooked freshwater fish consumption and eosinophilia prompted suspicion of occult liver fluke infection, despite negative initial stool tests. Diagnostic treatment with albendazole resulted in a decrease in WBC and eosinophil counts; subsequent detection of liver flukes in ENBD fluid and positive liver fluke IgG confirmed the diagnosis. At 2-month follow-up, all markers normalized. This case highlights the diagnostic value of eosinophilia in guiding the identification of parasitic infections in refractory post-ERCP inflammation, and the utility of diagnostic therapy when direct evidence is lacking.

## Introduction

Acute Suppurative Obstructive Cholangitis (AOSC) is a life-threatening biliary disorder characterized by biliary obstruction and suppurative infection (1, 2). It can rapidly progress to biliary hypertension, systemic inflammatory response syndrome (SIRS), and even multiple organ failure, thereby becoming a major cause of death in patients with biliary diseases. Clinically, Endoscopic Retrograde Cholangiopancreatography (ERCP) combined with biliary drainage serves as the cornerstone of AOSC treatment, as it effectively alleviates obstruction. For most patients, inflammatory markers and bilirubin levels gradually decline after surgery. However, a subset of patients presents with persistent abnormalities in bilirubin and leukocyte levels post-ERCP, accompanied by poor response to antibiotic therapy—indicating the existence of occult pathogenic factors.

In such cases, clinicians must broaden the diagnostic perspective, not limited to surgical complications and systematically rule out common etiologies to identify the underlying cause. Common triggers of biliary obstruction include calculi and tumor compression, while parasitic infection, though less prevalent, is easily overlooked due to its insidious early symptoms and the limited sensitivity of routine tests. This report describes an AOSC case in which the patient experienced prolonged illness after ERCP, attributed to occult liver fluke infection, a rare but clinically significant etiology in endemic regions or individuals with raw freshwater fish consumption.

## Case presentation

A 53-year-old male patient was admitted to the emergency department on April 10, 2025 (defined as “Day 0”), with complaints of “epigastric distension for 10 days and jaundice for 3 days”. Ten days prior to admission, he developed postprandial epigastric distension accompanied by anorexia, which he initially ignored. Subsequently, he gradually developed generalized skin and scleral jaundice, dark tea-colored urine, and intermittent fever (with a maximum temperature of 39 °C), which temporarily subsided after taking oral antipyretics.

**Figure 1:**
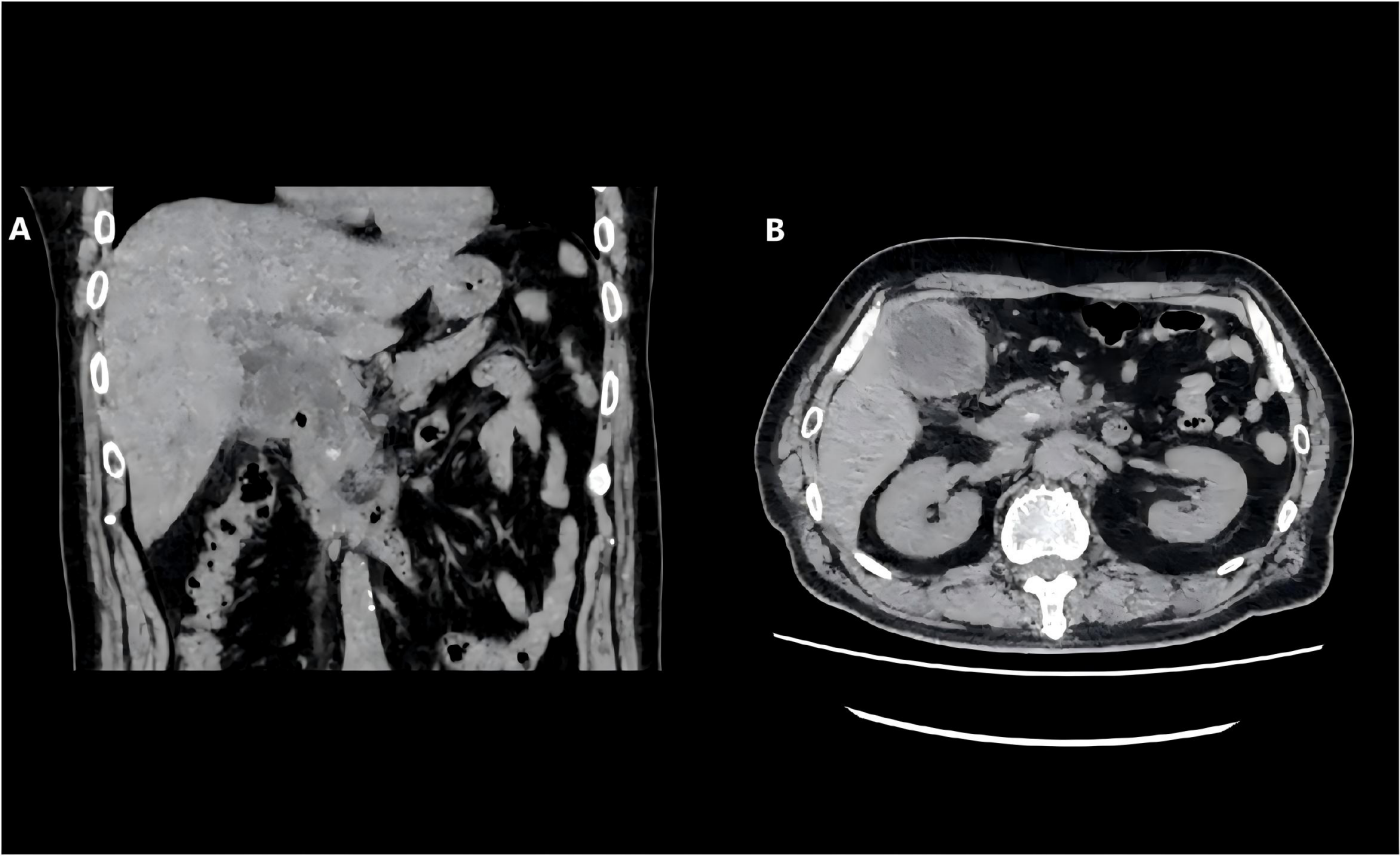
Preoperative CT scan indicated common bile duct stones, gallstones with cholecystitis. **(A)** Preoperative CT coronal image. **(B)** Preoperative CT axial image.

Emergency examinations revealed severe cholestasis (total bilirubin [TBIL] 185.2μmol/L, direct bilirubin [DBIL] 140.1μmol/L), significantly elevated inflammatory markers (white blood cell [WBC] count 10.52×10^9^/L, C-reactive protein [CRP] 113.73 mg/L), and a mild increase in eosinophils (0.97×10^9^/L). Imaging examinations showed a 0.8 cm calculus in the lower common bile duct (CBD), intrahepatic and extrahepatic bile duct dilatation, gallbladder calculi with cholecystitis, and pericholecystic inflammatory infiltration (**Figure 1A, B**). The patient had no significant past medical history, abnormal family history, or exposure to epidemic water. Physical examination revealed moderate skin and scleral jaundice. No epigastric tenderness, rebound tenderness, or Murphy’s sign was observed, and the liver and spleen were not palpable below the costal margin.

**Figure 2:**
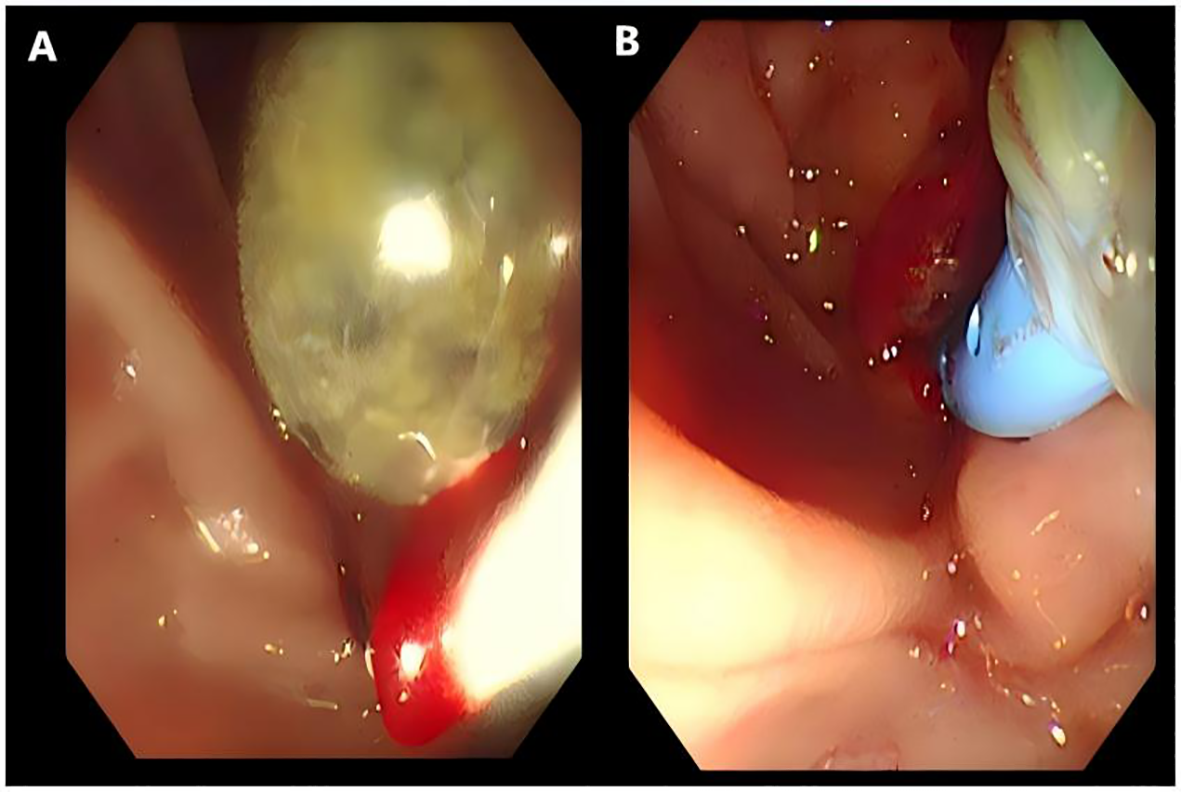
Images during ERCP surgery. **(A)** Removal of stones (0.8 × 0.6cm) from the lower segment of the common bile duct during ERCP surgery. **(B)** Placement of nasobiliary drainage during ERCP surgery.

On April 11(Day 1), the patient underwent ERCP. During the procedure, a calculus (0.8×0.6 cm) was successfully removed from the lower CBD (**Figure 2A**), and an Endoscopic Nasobiliary Drainage (ENBD) tube was placed (**Figure 2B**). Purulent bile was drained intraoperatively. Combined with preoperative clinical manifestations and examination results, a diagnosis of AOSC was confirmed. Postoperatively, standard antibiotic therapy was administered (ceftriaxone sodium-sulbactam sodium, 3 g, every 12 hours, for a 7-day course). However, follow-up tests showed no improvement: bilirubin remained significantly elevated (TBIL 180.7μmol/L, DBIL 145.1μmol/L), and inflammatory markers further increased (WBC 28.12×10^9^/L, CRP 35.90 mg/L).

**Figure 3:**
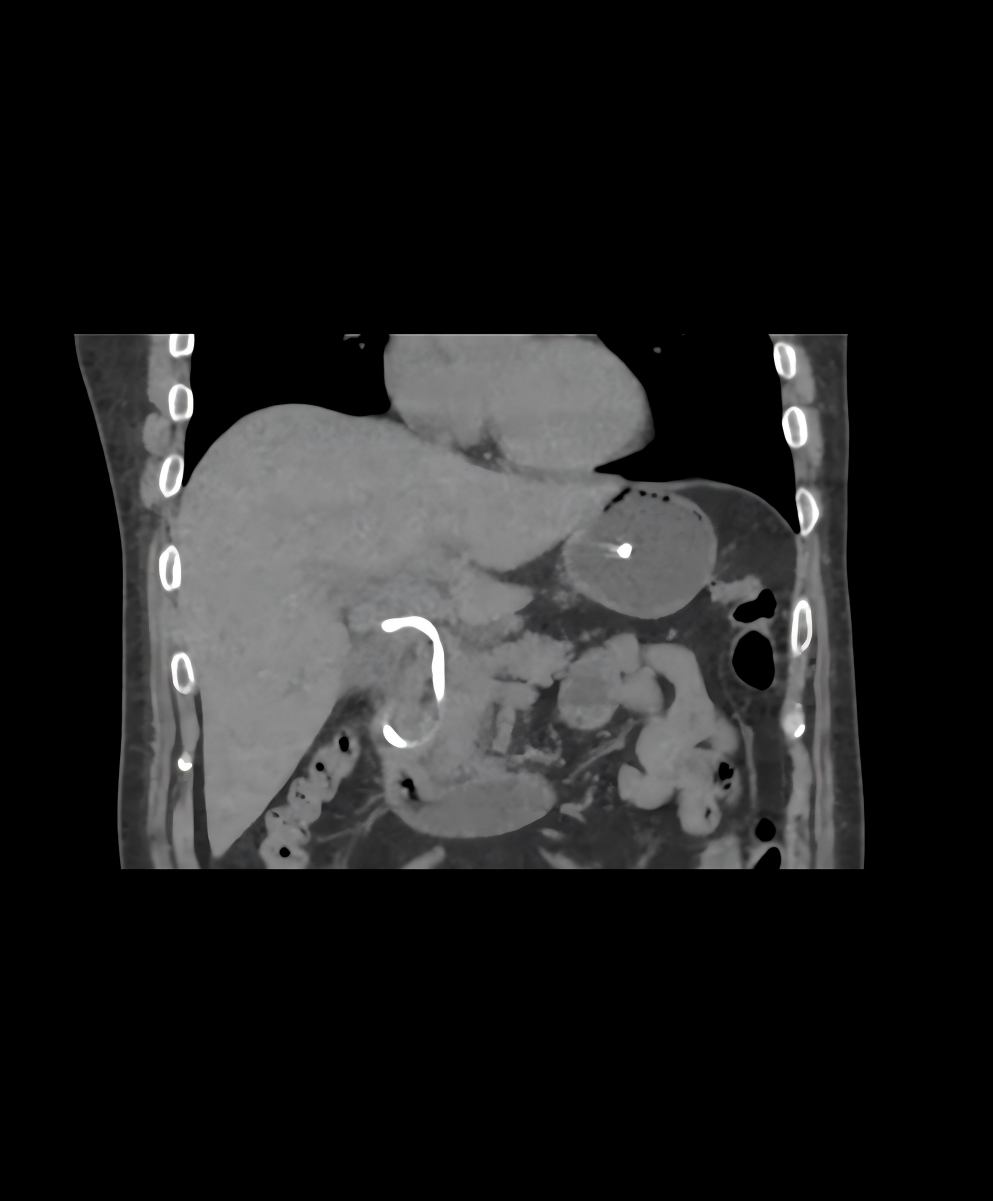
Postoperative follow-up CT scan showed the position of the nasal bile duct was normal, with no displacement or folding.

To identify the cause of persistent abnormal bilirubin and inflammatory markers post-ERCP, we launched a systematic investigation. Residual biliary calculi were ruled out by contrast-enhanced CT showing complete stone removal and unobstructed biliary drainage. By confirming the correct tube position (**Figure 3**) and stable drainage (with a drainage volume exceeding 300ml/day and no obvious turbidity or blockage in the drainage fluid), ENBD tube dysfunction was ruled out. Biliary neoplasms were ruled out by normal tumor markers and absence of space occupying lesions on imaging. Primary sclerosing cholangitis (PSC) was excluded based on normal alkaline phosphatase, negative autoimmune markers and no “beaded” biliary changes on post-ERCP CT.

After ruling out the above common etiologies, the team re-evaluated the trend of changes in laboratory indicators of the patient. At admission, eosinophils were only mildly elevated (0.97×10^9^/L). Although eosinophilia is a classic marker of parasitic infection, it can also be associated with allergic disorders, hematologic diseases, malignancies, certain rare infectious conditions, and eosinophilic gastroenteritis. Moreover, initial stool examination for parasite eggs was negative. Therefore, at that stage, the mild eosinophilia alone was insufficient to definitively point to parasitic etiology. However, post-ERCP, despite antibiotic therapy and resolution of biliary obstruction, eosinophils progressively increased to 20.26×10^9^/L. This marked elevation, in conjunction with the patient’s history of raw freshwater fish consumption and the exclusion of other common causes, prompted reconsideration of occult liver fluke infection. Further inquiry revealed a history of undercooked freshwater fish consumption. Although initial stool tests were negative, the combination of dietary history, persistent eosinophilia, and exclusion of common causes strongly suggested occult liver fluke infection. Diagnostic parasitic therapy was then initiated: albendazole (0.8g orally, once daily) combined with polyene phosphatidylcholine capsules (2 capsules orally, three times daily) for liver protection.

After receiving diagnostic therapy, the patient’s condition improved rapidly: recheck results showed that WBC decreased to 9.98×10^9^/L and eosinophils decreased to 5.26×10^9^/L. Subsequent etiological and immunological tests confirmed liver fluke IgG (+) and Toxoplasma gondii IgG (+), and brown liver fluke worms were detected in the ENBD drainage fluid. The final diagnosis was “acute suppurative obstructive cholangitis post-ERCP with occult liver fluke infection”.

**Figure 4:**
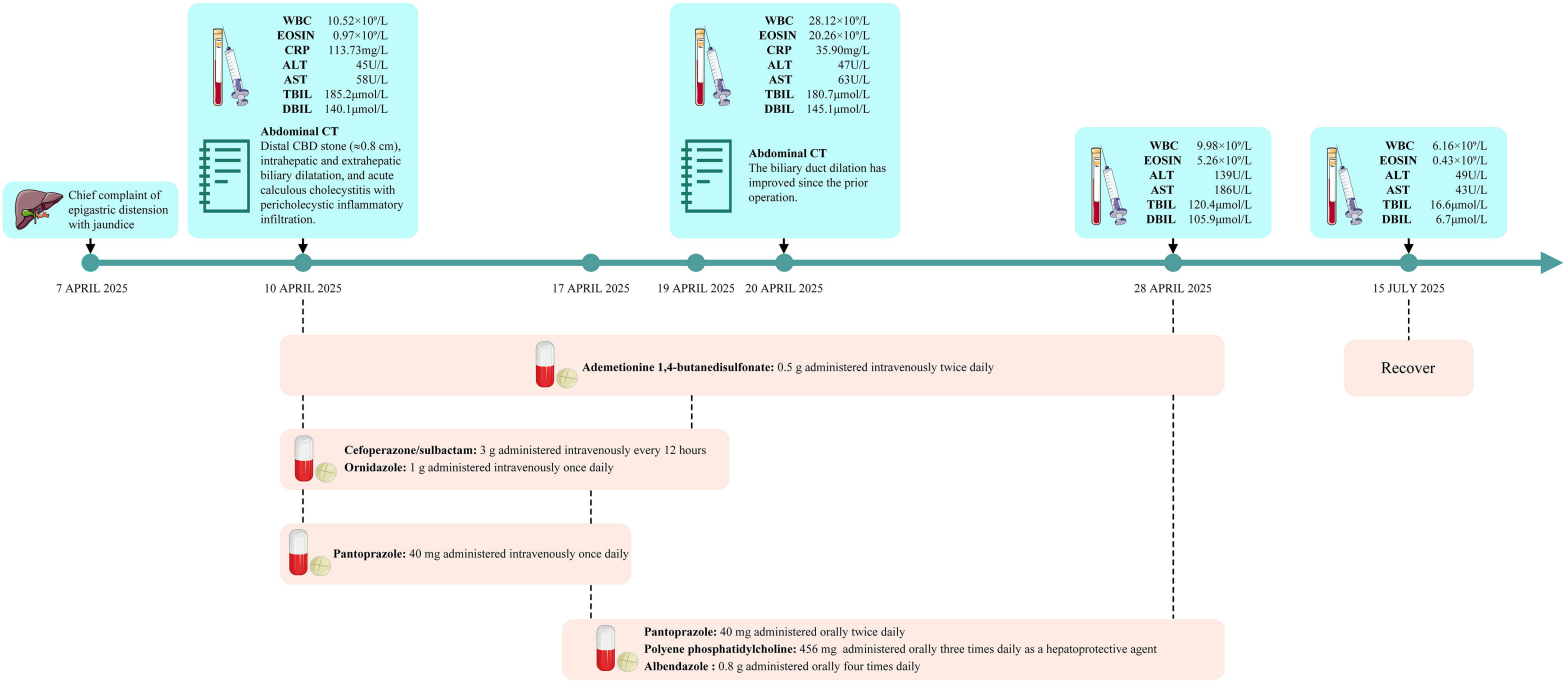
Information on medication and inspection results throughout the entire process.

After discharge, the patient continued to receive systematic deworming and liver/gallbladder protection treatment. At the 2-month follow-up outpatient visit, the patient had no recurrent symptoms or complications. He expressed satisfaction with the timely diagnosis and targeted treatment received. Laboratory tests showed normalized blood routine (WBC 6.16×10^9^/L, eosinophils 0.43×10^9^/L) and liver function. The medication and inspection results throughout the entire process are shown in **Figure 4**.

## Discussion

This case involves an AOSC patient complicated with occult liver fluke infection after ERCP. The diagnostic challenge lay in distinguishing parasitic infection from more common post-ERCP complications. While residual stones or drainage failure are typical causes of persistent inflammation (3), they were ruled out by imaging and clinical assessment. Biliary neoplasms were ruled out through normal tumor markers and the absence of space-occupying lesions on imaging. PSC was excluded based on normal ALP levels, negative immunological markers, and no “beaded” biliary strictures (4). The key diagnostic clue was eosinophilia, which prompted consideration of parasitic etiology despite negative stool tests.

Liver fluke infection represents a significant yet underrecognized global health burden. Globally, over 750 million people are at risk of liver fluke infection, with the highest burden concentrated in Southeast Asia and the Western Pacific Region. Three main species are responsible for human infection: Opisthorchis viverrini, O. felineus, and Clonorchis sinensis, all transmitted through the consumption of raw freshwater fish containing metacercariae. Geographically, O. viverrini is endemic in Vietnam, Laos, Thailand, and Cambodia, while C. sinensis infection is prevalent in rural areas of China and South Korea (5). Despite affecting millions of people worldwide, liver fluke infection has not been fully recognized in daily life and clinical practice. In this case, the patient’s dietary history of raw freshwater fish consumption and geographic background aligned with this endemic risk pattern, underscoring the importance of considering parasitic etiology in patients presenting with unexplained cholangitis, particularly those from or with travel to endemic regions.

The mechanism by which liver fluke infection causes prolonged inflammation post-ERCP is indirectly supported by the disease course. Adult liver flukes parasitize the biliary tract; even after calculus obstruction is relieved, the worms continue to damage the biliary mucosa through mechanical irritation and toxin secretion, leading to mucosal hyperemia and edema. Meanwhile, egg deposition may induce local inflammatory responses—this explains why inflammation persisted despite standardized antibiotic therapy (ordinary antibiotics have no effect on parasites, and inflammation can only be controlled by eliminating the worms). The use of diagnostic therapy proved valuable when direct evidence (detection of liver flukes in ENBD drainage fluid or positive liver fluke IgG) was lacking. The rapid clinical and laboratory response supported the diagnosis and avoided delays in treatment. This approach is particularly useful in settings where parasitic infections are suspected but initial tests are inconclusive.

This case provides three key clinical insights. First, for patients with persistent leukocytosis, eosinophilia, or bilirubin elevation post-ERCP, clinicians should avoid immediately attributing the issue to surgical complications. Instead, they should systematically rule out common etiologies using imaging and laboratory tests, and then explore other potential causes based on the patient’s individual exposure history (e.g., dietary or contact history). Second, elevated eosinophils should be recognized as an important indicator of parasitic infection—especially in patients with high-risk dietary histories (e.g., consumption of raw freshwater fish). Even if routine stool tests are negative, parasitic infection should be considered, and diagnostic therapy should be initiated when appropriate. Third, diagnostic therapy has value in the management of occult parasitic infections: when there is strong suspicion but no direct etiological evidence, favorable responses to targeted deworming therapy can serve as important diagnostic evidence, preventing treatment delays while waiting for confirmatory etiological results.

In terms of limitations, the sensitivity of our initial diagnostic method (fecal parasite egg detection) is insufficient, resulting in delayed diagnosis of parasitic infections, which may exacerbate inflammation and prolong the process. Fecal testing may result in missed diagnosis due to low ovulation levels of parasites, leading to delayed identification of biliary mucosal inflammation related to parasitic infections. In addition, routine anti infective treatment during the delayed diagnosis period is ineffective against parasites, which may lead to persistent abnormalities in postoperative inflammatory indicators (WBC, eosinophils), prolong the patient’s treatment period, and pose a potential risk of biliary tract injury.

## Conclusions

Systematic etiological investigation and diagnostic therapy identified liver fluke infection as the cause of prolonged inflammation post-ERCP in this case. The diagnostic process highlights the importance of considering parasitic infection in patients with unexplained post-ERCP inflammation, especially those with eosinophilia and relevant dietary history. The combination of clinical suspicion, diagnostic therapy, and confirmatory testing enabled accurate diagnosis and effective treatment.

## Data Availability

The original contributions presented in the study are included in the article/supplementary files. Further inquiries can be directed to the corresponding author.
